# Electrospun Eco-Friendly Materials Based on Poly(3-hydroxybutyrate) (PHB) and TiO_2_ with Antifungal Activity Prospective for Esca Treatment

**DOI:** 10.3390/polym12061384

**Published:** 2020-06-20

**Authors:** Mariya Spasova, Olya Stoilova, Nevena Manolova, Iliya Rashkov, Mladen Naydenov

**Affiliations:** 1 Laboratory of Bioactive Polymers, Institute of Polymers, Bulgarian Academy of Sciences, Acad. G. Bonchev St, bl. 103A, BG-1113 Sofia, Bulgaria; stoilova@polymer.bas.bg (O.S.); manolova@polymer.bas.bg (N.M.); rashkov@polymer.bas.bg (I.R.); 2 Department of Microbiology, Agricultural University, BG-4000 Plovdiv, Bulgaria; mladen@au-plovdiv.bg

**Keywords:** antifungal activity, TiO_2_, electrospinning, electrospraying, PHB, *Phaeomoniella chlamydospora*, esca

## Abstract

Esca is a type of grapevine trunk disease that severely affects vine yield and longevity. *Phaeomoniella chlamydospora* (*P. chlamydospora*) is one of the main fungi associated with esca. The aim of the present study was to obtain eco-friendly materials with potential antifungal activity against *P. chlamydospora* based on biodegradable and biocompatible poly(3-hydroxybutyrate) (PHB), nanosized TiO_2_-anatase (nanoTiO_2_), and chitosan oligomers (COS) by conjunction of electrospinning and electrospraying. One-pot electrospinning of a suspension of nanosized TiO_2_ nanoparticles in PHB solution resulted in materials in which TiO_2_ was incorporated within the fibers (design type “*in*”). Simultaneous electrospinning of PHB solution and electrospraying of the dispersion of nanosized TiO_2_ in COS solution enabled the preparation of materials consisting of PHB fibers on which TiO_2_ was deposited on the fibers’ surface (design type “*on*”). Several methods including scanning electron microscopy (SEM), transmission electron microscopy (TEM), Fourier transform infrared spectroscopy (FT-IR), X-ray diffraction analysis (XRD), thermogravimetric analyses (TGA) and water contact angle were utilized to characterize the obtained materials. The incorporation of nanoTiO_2_ in the PHB fibers, as well as nanoTiO_2_ deposition onto the surface of the PHB fibers resulted in increased roughness and hydrophobicity of the obtained composite fibrous materials. Moreover, TiO_2_-*on*-PHB fibrous material exhibited complete inhibition of fungal growth of *P. chlamydospora*. Therefore, the obtained eco-friendly fibrous materials based on PHB and nanoTiO_2_ are promising candidates for protection against esca in agriculture.

## 1. Introduction

Esca is a disease of grapevines that causes trunk damage and entire-plant wilting and has been known about since ancient times [1,2,3]. This grapevine disease is caused mainly by *Phaeoacremonium chlamydospora* and *Phaeoacremonium aleophilum* species [4,5]. Over the last three decades, the impact of esca disease has become an issue of great importance. Symptoms of the disease manifest in severe or chronic forms, which may affect the entire plant or individual canes of the same plant [1,6]. In addition, the fruits can be affected as well [7]. The wounds formed on vines during the pruning procedure are found to be the main point of penetration by *P. chlamydospora* and *P. aleophilum* spores in grapevines [3,8]. Up to now, sodium arsenite is the only known and effective agent for combating esca. However, arsenic and its compounds have been classified as carcinogenic and currently are not allowed for use [9]. Therefore, it cannot be applied for the production of plant protection products. To date, there are no curative approaches to fight against esca, and only preventative methods are applied [10]. Treatments with hot water are sometimes used to obtain commercial plants with good sanitary conditions. This process is generally performed at 50 °C for 30 min; however, it is stressful for the plants [11].

Nanotechnologies are utilized in the design, characterization, production, and application of new generations of materials, structures, devices, and systems, having one or more dimensions of about 100 nanometers or less, and therefore possessing unique size-related properties. Nanotechnology has the potential to solve diverse problems with significant social and economic impacts. For instance, electrospinning allows simple and versatile preparation of polymer fibers with micro- and nanosized diameters, with extremely long lengths, up to several meters, and with unique properties—large specific surface area and fine porous structure [12,13]. In our previous study, we showed that electrospinning could be easily applied for the preparation of fibers containing chitosan and *Trichoderma viride* spores for agricultural crop protection. The prepared biohybrid fibrous mats inhibited the growth of *Fusarium* and *Alternaria* strains. Moreover, the facile covering of different plant parts by direct electrospinning was demonstrated [14].

Soy protein, polyvinyl alcohol and polycaprolactone have been electrospun on rayon membranes in order to obtain a material that hinders the penetration of fungal spores [15]. However, physical blocking has proven unsatisfactory and the inclusion of a component with antifungal activity has been put forward. Therefore, one recent report has been published on the use of electrospun materials from poly(lactide-*co*-glycolide) and poly(butyleneadipate-*co*-terephthalate), and it incorporated an antifungal agent, polyhexamethylene guanidine, as bandages to prevent esca disease by blocking the penetration of *P. chlamydospora* spores [16]. In addition, the fibrous membranes prepared by electrospinning enable air and moisture permeation, allowing a plant wound to “breathe”. The authors reported, however, that further optimization was needed to find more efficient polymers and a more appropriate choice of antifungal additives.

In the present study, we propose the facile fabrication of fibrous materials containing an easily available, naturally occurring compound with antifungal activity—titanium dioxide. Titanium dioxide is one of the most extensively studied, and used, metal oxide with photocatalytic and antibacterial properties [17,18]. Moreover, TiO_2_ (anatase and rutile) is well known for its ability to produce reactive oxygen species [19]. Recently, we demonstrated that nanostructured composite materials containing TiO_2_ nanoparticles evoke particular interest due to their potential applications in biomedicine, biotechnologies, and for environmental protection [20,21].

The aim of the present study was to prepare nanostructured composite mats with diverse design based on PHB and nanoTiO_2_ by electrospinning, or in combination with electrospraying. The effect of the composition and material design on morphology and wetting was investigated. Moreover, we assessed the antifungal activity of the prepared electrospun materials using a model fungus, *Phaeoacremonium chlamydospora*, which is one of the main species associated with esca disease. We have determined that the proposed approach is very promising for plant protection against esca.

## 2. Materials and Methods

### 2.1. Materials

Poly(3-hydroxybutyrate) (PHB, 330,000 g/mol, Biomer, Schwalbach, Germany), titanium (IV) oxide (nanoTiO_2_) (99.7% anatase nanopowder, <25 nm, Sigma–Aldrich, St. Louis, MO, USA) and chitosan oligomers (COS, average molecular weight 3000–5000 g/mol, Kitto Life Co. LTD, Korea) were used. *N*,*N*-dimethylformamide (DMF), chloroform (CHCl_3_) and ethanol were delivered from Merck (Darmstadt, Germany) and used as received. Potato dextrose agar medium was purchased also from Merck (Darmstadt, Germany). Disposable consumables were supplied by Orange Scientific, Braine-l’Alleud, Belgium.

### 2.2. Fabrication of Fibrous Materials

**Figure 1:**
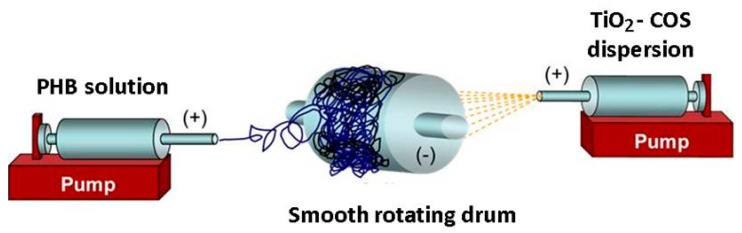
Schematic diagram of the electrospinning/electrospraying setup.

Fibrous PHB, TiO_2_-*in*-PHB and TiO_2_-*on*-PHB materials were fabricated by electrospinning or by simultaneous electrospinning and electrospraying, as described in detail elsewhere [21]. In brief, prior to electrospinning, the PHB spinning solution (14% *w*/*v*) in CHCl_3_/DMF (4/1 *v*/*v*) was obtained by heating (60 °C) using a reflux condenser. PHB fibrous materials were prepared by direct electrospinning of PHB solution. TiO_2_-*in*-PHB fibrous materials were obtained by electrospinning of a mixture of PHB solution with nanoTiO_2_ (7% *w*/*v*). The obtained PHB solution and nanoTiO_2_/PHB mixture (total nanoTiO_2_ concentration 33 wt.%) were placed in syringes and delivered (NE-300, New Era Pump Systems, Inc.) at a constant feeding rate of 3 mL/h and 5 mL/h, respectively. Electrospinning was performed at a voltage of 25 kV, tip-to-collector distance of 25 cm and 1500 rpm collector rotation speed. TiO_2_-*on*-PHB fibrous materials were prepared using a PHB spinning solution (14% *w*/*v*) for electrospinning and nanoTiO_2_-COS dispersion for electrospraying. For this purpose, an aqueous COS solution (0.5%) was added to the nanoTiO_2_ (10% *w*/*v*) dispersion in ethanol. The prepared (nanoTiO_2_-COS) dispersion in ethanol/water (4/1 *v*/*v*) was sonicated (Bandelin Sonorex, 160/640 W, 35 kHz) for 1 h. Then, the PHB spinning solution and the prepared nanoTiO_2_-COS dispersion were placed in two separate syringes. The syringes were positioned at an angle of 180° relative to each other. A rotating drum collector (1500 rpm) was used. Schematic representation of the electrospinning/electrospraying setup is shown in Figure 1. A high voltage of 25 kV was applied to the both needles. The PHB solution was fed at a rate of 3 mL/h, and that of nanoTiO_2_-COS at 2 mL/h. The distance from the tip to collector was 25 and 10 cm for electrospinning and electrospraying, respectively.

### 2.3. Characterization of the Fibrous Composite Materials

The morphology of the materials was observed by scanning electron microscopy (SEM). The samples were vacuum-coated with carbon and analyzed by a Philips 515 SEM (Tokyo, Japan). The mean fiber diameter was determined by using ImageJ software [22]. The criteria for overall evaluation of electrospun materials were applied [23]. Transmission electron microscopy (TEM) was conducted on a JEM 2100 (JEOL Co. Ltd., Freising, Germany) operating at a voltage of 200 kV. Samples were prepared by direct depositing on a copper grid.

Static contact angle measurements of the fibrous materials were performed using an Easy Drop DSA20E Krűss GmbH drop shape analysis system (Hamburg, Germany) at 20 ± 0.2 °C. A sessile drop of deionized water with a volume of 5 μL, controlled by a computer dosing system, was deposited onto the fibrous materials. The contact angles were calculated by computer analysis of the acquired images of the droplet. The data were averaged from 20 measurements for each sample.

X-ray diffraction (XRD) analyses were performed using a computer-controlled D8 Bruker Advance diffractometer with filtered Cu Kα radiation and a LynxEye detector at room temperature. Data were collected in the 2θ range from 5.3° to 80° with a step of 0.02° and counting time of 1 s step^−1^. Diffrac*plus* EVA using the ICDD-PDF2 database was used for phase identification.

Fourier-transform infrared (FT-IR) spectroscopy analyses were performed using an IRAffinity-1 spectrophotometer (Shimadzu Co., Kyoto, Japan), supplied with a MIRacle ATR device (diamond crystal; depth of penetration of the IR beam into the sample—approximately 2 μm) (PIKE Technologies, Madison, WI, USA) in the range of 600–4000 cm^−1^ with a resolution of 4 cm^−1^. All spectra were corrected for H_2_O and CO_2_ using the IRsolution software program.

Thermogravimetric analyses (TGA) were carried out in the 25–1000 °C range at 10 °C/min rate under nitrogen with a TA Instruments Q500 (HiRes method).

### 2.4. In Vitro Antifungal Assay

The fungi *P. chlamydospora* CBS 239.74 (Westerdijk Fungal Biodiversity Institute, Utrecht, The Netherlands) were used in the in vitro antifungal assay. For the preparation of the conidia suspension, test microorganisms were grown on potato dextrose agar (PDA) medium for 14 days.

Conidia were obtained by pouring 5 mL of sterile water onto the plate and washing off with a sterile loop. Conidia suspensions were filtered through two layers of sterile round cloth to remove mycelial fragments. The final concentration of conidia was adjusted to 10^7^ conidia/mL with sterile water. The fibrous materials were cut in disks with diameters of 4.5 cm and thickness ~1 µm. Digital Thickness Gauge FD 50 (Käfer GmbH, Villingen-Schwenningen, Germany) was used to determine the thickness of the fibrous materials. All fibrous materials were sterilized for 30 min under UV light in the laminar box before being used for further experiments. Then, the fibrous material (in a disk form) was placed between the two parts of the filtration device and supplied with a pump. The two parts of the device were pinched with a clip. After that, 20 mL of spore conidia suspension was passed through each type the fibrous material. Then, every used disk was taken with pincers and placed on a surface of a solid PDA medium in a Petri dish. The Petri dishes were placed for 96 h at 28 °C under light irradiation by a 10 W lamp at wavelength of 420 nm. Then, the fungal growth was assessed. The concentration of conidia, passed through the materials, was determined using a hemocytometer.

## 3. Results and Discussion

**Figure 2:**
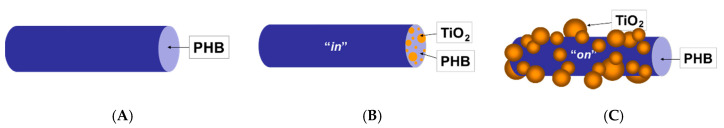
Schematic representation of fibers: (**A**) PHB, (**B**) TiO_2_-*in*-PHB and (**C**) TiO_2_-*on*-PHB.

Three types of fibrous materials based on biocompatible and biodegradable PHB and nanosized TiO_2_-anatase were fabricated by electrospinning alone or in conjunction with electrospraying (Figure 2).

**Figure 3:**
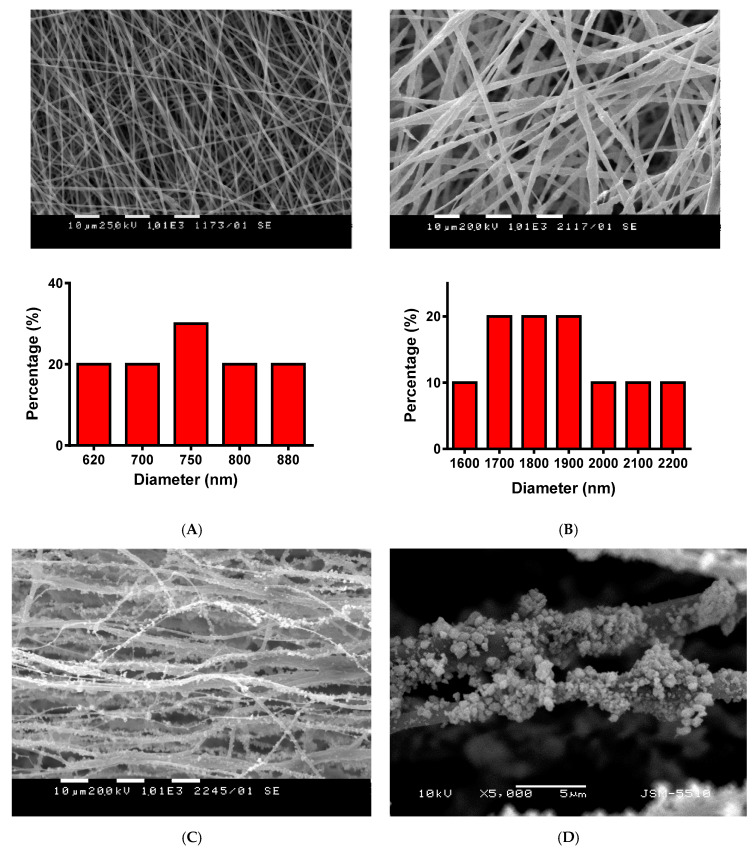
SEM micrographs of fibrous materials: (**A**) PHB (×1000), (**B**) TiO_2_-*in*-PHB (×1000), (**C**) TiO_2_-*on*-PHB (×1000) and (**D**) TiO_2_-*on*-PHB (×5000). The fiber diameter distributions are given for (**A**) and (**B**).

**Figure 4:**
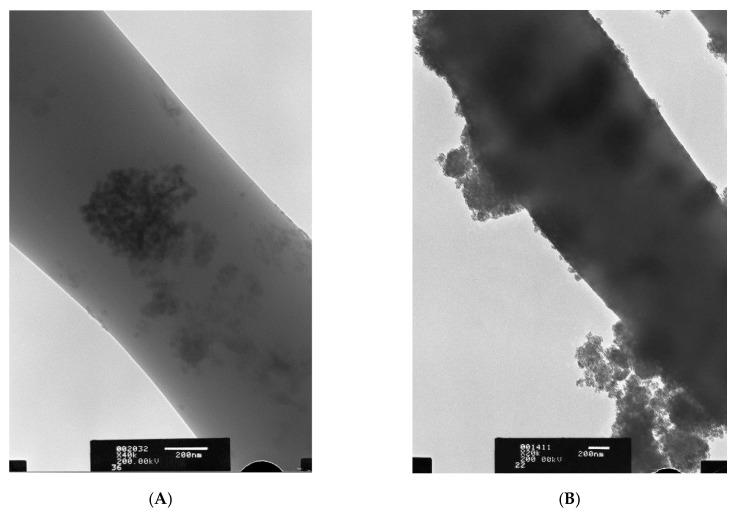
TEM images of fibers: (**A**) TiO_2_-*in*-PHB and (**B**) TiO_2_-*on*-PHB.

SEM micrographs and fiber diameter distributions of the prepared PHB, TiO_2_-*in*-PHB and TiO_2_-*on*-PHB electrospun materials are shown in Figure 3. It can be clearly seen that the selected conditions for electrospinning resulted in the fabrication of uniform and defect-free PHB fibers. The electrospinning of PHB solution resulted in the preparation of continuous fibers with a mean fiber diameter of 750 ± 130 nm (Figure 3A). Adding nanoTiO_2_ to the PHB solution resulted in an increase in the mean fiber diameter to 1900 ± 300 nm. It was found that incorporation of nanoTiO_2_ resulted in the preparation of fibers with a rough surface (Figure 3B and Figure 4A). Moreover, it could be easily seen that some of the TiO_2_ particles are very close to the fiber surface. This morphological alteration of the type “*in*” materials is a result of the incorporation of nanoTiO_2_ particles in the fibers. It was detected that part of the particles aggregated during the electrospinning. Representative SEM images of the obtained TiO_2_-*on*-PHB fibrous materials are shown in Figure 3C,D. It can be seen that electrospraying of the dispersions containing nanoTiO_2_-COS resulted in the decoration of the surface of the PHB fibers with nanoTiO_2_. The presented SEM at high magnification showed that the TiO_2_ particles possessed spherical form with small (ca. 20 nm) and large (500 nm) diameters (Figure 3D). Apparently, COS stabilized the nanoTiO_2_ dispersion and served as a sticking agent for TiO_2_ onto the PHB fibers.

The distribution of TiO_2_ nanoparticles in/on the PHB fibers, depending on the materials type, was observed by TEM. As seen from the presented TEM micrograph of TiO_2_-*in*-PHB, the nanoTiO_2_ particles were distributed mainly in the bulk of the PHB fibers (Figure 4A). Some of the nanoparticles formed agglomerates. Electrospraying of TiO_2_-COS dispersion on the PHB fibers resulted in enrichment of the fiber surface in the nanoTiO_2_ particles (Figure 4B). Detailed observation of the TiO_2_-*on*-PHB materials using SEM and TEM revealed that all the fibers were decorated with TiO_2_ particles.

The FT-IR spectra of the PHB, TiO_2_-*in*-PHB and TiO_2_-*on*-PHB fibrous materials (Figure S1) showed bands characteristic of PHB with the carbonyl stretching band at 1721 cm^−1^ and the C‒O stretching band at 1055 cm^−1^. Clearly, no interaction between PHB and nanoTiO_2_ was detected in the FT-IR spectra of TiO_2_-*in*-PHB and TiO_2_-*on*-PHB fibrous materials.

**Figure 5:**
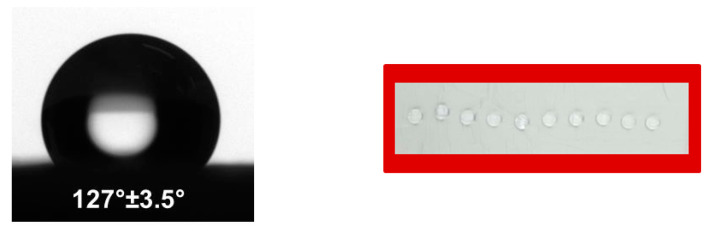
Water droplets (5 µL) deposited on the TiO_2_-*on*-PHB mat.

It is known that the affinity to water of the host surface affects the adhesion and growth of fungal plant pathogens [24]. For this reason, it is important to determine the water contact angle of the prepared fibrous materials that will be in contact with fungal species. The water contact angle of neat PHB fibrous materials was a 108° ± 3.3°, i.e., this fibrous material was hydrophobic. The incorporation of nanoTiO_2_ in the fibers resulted in the preparation of rough fibers, and the roughness led to the increase of contact angle value in comparison with the neat PHB fibers. The measured water contact angles for TiO_2_-*in*-PHB and TiO_2_-*on*-PHB fibrous materials were 124° ± 2.3° and 127° ± 3.5° (Figure 5), respectively. The structure of TiO_2_-*on*-PHB fibrous materials resembled a lotus leaf architecture and possessed a rougher surface compared with that of the TiO_2_-*in*-PHB fibrous materials. However, the measured water contact angle values for the TiO_2_-*in*-PHB and TiO_2_-*on*-PHB fibrous materials were very close. This finding could be explained by the effect of COS, which decreased the water repellency and this effect could not be compensated by the higher surface roughness of the type “*on*” materials.

**Figure 6:**
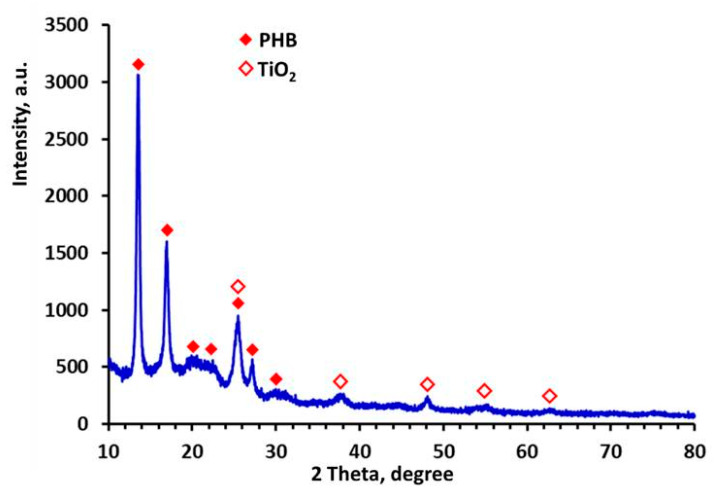
XRD pattern of fibrous materials: TiO_2_-*in*-PHB.

The crystallinity of the PHB and the crystal phase of the nanoTiO_2_ were analyzed by X-ray diffraction analysis. It was of interest to determine if TiO_2_ incorporated in the PHB fibers was detectable with XRD analysis. In the XRD patterns of TiO_2_-*in*-PHB (Figure 6), characteristic diffraction peaks of PHB positioned at 13.5° (020), 16.9° (110), 20.1° (021), 21.5° (101), 25.5° (111) and 27.2° (040) were observed. In addition, the TiO_2_ anatase phase with peaks at 25.5° (101), 37.6° (004), 48.2° (200), 56.3° (211) and 62.8° (204) was also identified. The obtained results were in agreement with TiO_2_ (anatase) standard data [25]. Characteristic diffraction peaks of TiO_2_ anatase and PHB were detected in the XRD spectra of TiO_2_-*on*-PHB as well (figure not shown).

**Figure 7:**
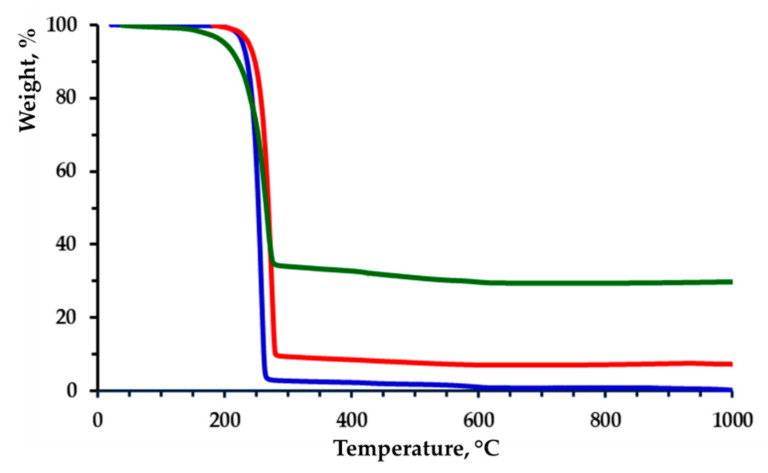
TGA thermograms of fibrous PHB (blue line), TiO_2_-*in*-PHB (green line) and TiO_2_-*on*-PHB (red line) materials.

The thermal stability of the fibrous materials was studied by TGA (Figure 7). In general, there were no significant differences in the thermal stability of the three types of materials. The most considerable decomposition occurred in the temperature range 250–270 °C due to the thermal destruction of the PHB. The residual weight was close to the weight of TiO_2_ in the feed.

**Figure 8:**
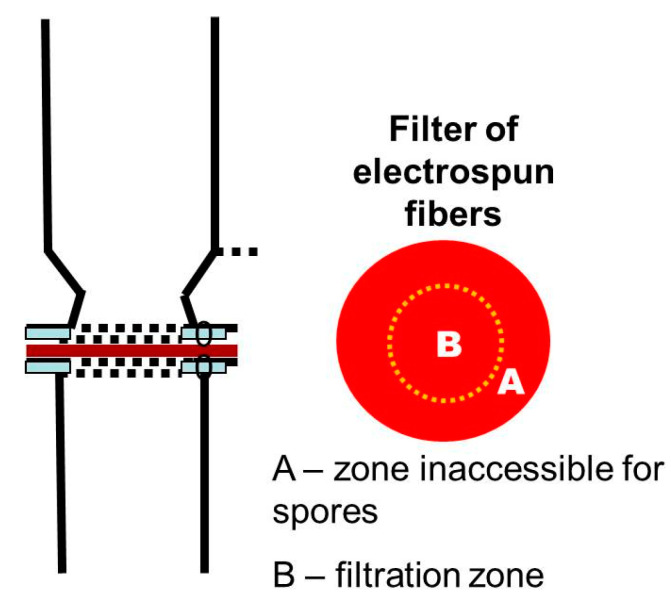
Schematic presentation of a filter position.

Recently, there has been a rising demand for developing novel plant protective agents and materials that are efficient and non-toxic. Nanostructured TiO_2_ is an environmentally friendly material and the prospects for its application in agriculture have already attracted attention. Up to now, TiO_2_ has been applied to degrade pesticides, for water purification, for pest and disease control, for plant growth, and for the detection of pesticides [20,26]. It is well known that pruning wounds are the main point of entry for a spore invasion of vine plants. The antifungal activity of the fibrous materials against *P. chlamydospora* was determined by performing microbiological tests. Initially, the barrier efficacy of PHB, TiO_2_-*in*-PHB and TiO_2_-*on*-PHB fibrous materials was studied. For that purpose, 20 mL of conidia suspension was passed through each fibrous material using a filtration device. A schematic presentation of the filter position in the device is shown in Figure 8. As can be seen, the conidia suspension contacted with the central part of the filter.

**Figure 9:**
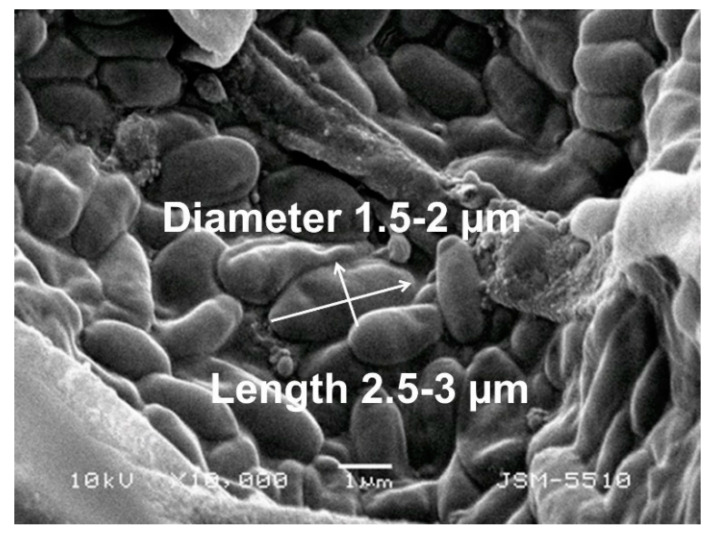
SEM micrograph of the *P. chlamydospora* conidia.

In the preliminary experiments, we determined the conidia size in the fungal suspension using SEM analysis. A SEM micrograph of the *P. chlamydospora* conidia used in the present study is shown in Figure 9. The size of the conidia was measured from the SEM micrograph with ImageJ software. It was determined that their diameters were ~1.5–2 µm and their lengths were ca. 2.5–3 µm.

**Figure 10:**
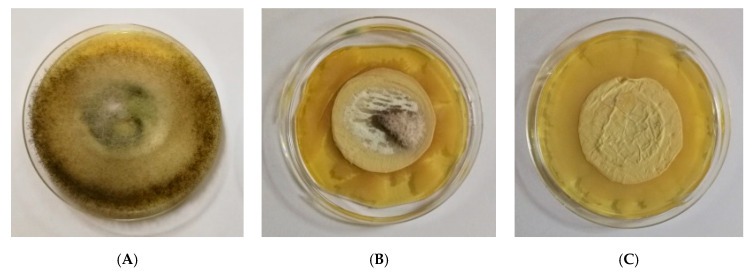
Digital images of the growth *P. chlamydospora* on the fibrous materials: (**A**) PHB, (**B**) TiO_2_-*in*-PHB and (**C**) TiO_2_-*on*-PHB.

The initial concentration in the filtration experiments was 1 × 10^7^ conidia/mL. It was found that in all cases, after passing through the fibrous materials, the conidia concentration decreased. The final spore concentrations were 5.7 × 10^4^, 3.3 × 10^3^ and 1.4 × 10^3^ for the PHB, TiO_2_-*in*-PHB and TiO_2_-*on*-PHB materials, respectively. We assumed that the higher roughness and the presence of protuberances in TiO_2_-*in*-PHB and TiO_2_-*on*-PHB fibrous materials led to more difficult passage of the conidia through them and resulted in a higher filtration efficiency compared to the PHB fibrous material. However, none of the used fibrous materials reached 100% conidia filtration efficiency. Then, the disks used in filtration experiments were taken off from the filtration device and were placed on a surface of a solid agar in a Petri dish. The Petri dishes were incubated for 96 h at 28 °C under light irradiation, and then the fungal growth was assessed. Digital images of growth of *P. chlamydospora* on the fibrous materials surface are shown in Figure 10. As can be easily seen, the PHB fibrous material used in the filtration experiments developed colonies of *P. chlamydospora* on its surface. Moreover, the fungi invaded the surfaces of all Petri dishes (Figure 10A). Electrospinning of mixed TiO_2_/PHB dispersion resulted in the preparation of materials with some antifungal properties (Figure 10B). Nevertheless, there was some fungal growth on the TiO_2_-*in*-PHB surface, especially in the central zone of the material, which was accessible by the conidia during the filtration experiment. However, they were comparatively much less than those observed on the surface of the PHB fibrous material. Interestingly, the simultaneous electrospinning of PHB solution and electrospraying of nanoTiO_2_-COS dispersion resulted in the preparation of a fibrous material with antifungal activity. The TiO_2_-*on*-PHB fibrous material that contacted with *P. chlamydospora* during filtration experiment showed complete inhibition of the growth of the fungi remaining in the fibrous material after the filtration (Figure 10C). The observation of a wide zone of inhibition around the TiO_2_-*on*-PHB materials evidenced that the nanoTiO_2_-COS deposited onto the surface of the PHB fibers imparted antifungal activity to the obtained electrospun material. The antifungal activity of the TiO_2_-*on*-PHB fibrous material could be due to the synergic action of TiO_2_ nanoparticles that are able to produce significant reactive oxygen species (ROS) [27] and the effect of chitosan oligomers. There are data in the literature showing that chitosan possesses antifungal activity against many fungal species [28]. Most probably, the antifungal activity of chitosan is due to its polycationic nature. We hypothesize that the positive charge of chitosan could interact with the negatively charged phospholipid parts of the fungal membrane. This will cause the leakage of cellular contents, thus leading to cell death.

## 4. Conclusions

Nanostructured fibrous materials of PHB, TiO_2_-*in*-PHB and TiO_2_-*on*-PHB were fabricated by electrospinning or in conjunction with electrospraying. The incorporation of nanoTiO_2_ into the PHB fibers or the decoration of PHB fibers with nanoTiO_2_-COS resulted in increased roughness and hydrophobicity of the obtained composite materials and imparted a considerable antifungal activity against *P. chlamydospora*. Complete suppression of the fungal growth was obtained in the case of the TiO_2_-*on*-PHB material. Thus, the obtained composite TiO_2_/PHB fibrous materials are promising for application in agriculture as eco-friendly materials for plant protection against the penetration and growth of the main causative fungi causing esca disease.

## Supplementary Materials

The following are available online at https://www.mdpi.com/2073-4360/12/6/1384/s1, Figure S1: IR spectra of fibrous materials and nanoTiO_2_.

## References

[1] Mugnai L., Graniti A., Surico G. (1999). Esca (black measles) and brown wood-streaking: Two old and elusive diseases of grapevines. Plant Dis..

[2] Hofstetter V., Buyck B., Croll D., Viret O., Couloux A., Gindro K. (2012). What if esca disease of grapevine were not a fungal disease?. Fungal Divers..

[3] Graniti A., Surico G., Mugnai L. (2000). Esca of grapevine: A disease complex or a complex of diseases?. Phytopathol. Mediterr..

[4] Larignon P., Dubos B. (1997). Fungi associated with esca disease in grapevine. Eur. J. Plant Pathol..

[5] Crous P., Grams W. (2000). *Phaeoemoniella chlamydospora* gen. et comb. Nov., a causal organism of Petri grapevine decline and esca. Phytopathol. Mediterr..

[6] Kuntzmann P., Villaume S., Larignon P., Bertsch C. (2010). Esca, BDA and Eutypiosis: Foliar symptoms, trunk lesions and fungi observed in diseased vinestocks in two vineyards in Alsace. Vitis.

[7] Bertsch C., Ramírez-Suero M., Magnin-Robert M., Larignon P., Chong J., Abou-Mansour E., Spagnolo A., Clément C., Fontaine F. (2013). Grapevine trunk diseases: Complex and still poorly understood. Plant Pathol..

[8] Eskalen A., Gubler W. (2001). Association of spores of *Phaeomoniella chlamydospora*, *Phaeoacremonium inflatipes*, and Pm. aleophilum with grapevine cordons in California. Phytopathol. Mediterr..

[9] Ciancio A., Mukerji K. (2008). Integrated Management of Diseases Caused by Fungi, Phytoplasma and Bacteria.

[10] Gramaje D., Armengol J. (2011). Fungal trunk pathogens in the grapevine propagation process: Potential inoculum sources, detection, identification, and management strategies. Plant Disease.

[11] Waite H., Gramaje D., Whitelaw-Weckert M., Torley P., Hardie W. (2013). Soaking grapevine cuttings in water: A potential source of cross contamination by micro-organisms. Phytopathol. Mediterr..

[12] Wen P., Zong M., Linhardt R., Feng K., Wu H. (2017). Review: Electrospinning: A novel nano-encapsulation approach for bioactive compounds. Trends Food Sci. Tech..

[13] Ahmed F., Lalia B., Hashaikeh R. (2015). A review on electrospinning for membrane fabrication: Challenges and applications. Desalination.

[14] Spasova M., Manolova N., Naydenov M., Kuzmanova J., Rashkov I. (2011). Electrospun biohybrid materials for plant biocontrol containing chitosan and *Trichoderma viride* spores. J. Bioact. Compat. Pol..

[15] Sett S., Lee M., Weith M., Pourdeyhim B., Yarin A. (2015). Biodegradable and biocompatible soy protein/polymer/adhesive sticky nano-textured interfacial membranes for prevention of esca fungi invasion, into pruning cuts and wounds of vines. J. Mater. Chem. B..

[16] Buchholz V., Molnar M., Wang H., Reich S., Agarwal S., Fischer M., Greiner A. (2016). Protection of vine plants against esca disease by breathable electrospun antifungal nonwovens. Macromol. Biosci..

[17] Nagalakshmi M., Karthikeyan C., Anusuya N., Brundha C., Jothi Basu M., Karuppuchamy S. (2017). Synthesis of TiO_2_ nanofiber for photocatalytic and antibacterial applications. J. Mater. Sci. Mater. Electron..

[18] Pant H., Pandeya D., Nam K., Baek W., Hong S., Kim H. (2011). Photocatalytic and antibacterial properties of a TiO_2_/nylon-6 electrospun nanocomposite mat containing silver nanoparticles. J. Hazard. Mater..

[19] Guo Y., Cheng C., Wang J., Wang Z., Jin X., Li K., Kang P., Gao J. (2011). Detection of reactive oxygen species (ROS) generated by TiO_2_(R), TiO_2_(R/A) and TiO_2_(A) under ultrasonic and solar light irradiation and application in degradation of organic dyes. J. Hazard. Mater..

[20] Korina E., Stoilova O., Manolova N., Rashkov I. (2014). Poly(3-hydroxybutyrate)-based hybrid materials with photocatalytic and magnetic properties prepared by electrospinning and electrospraying. J. Mater. Sci..

[21] Korina E., Stoilova O., Manolova N., Rashkov I. (2013). Multifunctional Hybrid Materials From poly(3-Hydroxybutyrate), TiO_2_ Nanoparticles, and chitosan oligomers by combining electrospinning/electrospraying and impregnation. Macromol. Biosci..

[22] Rasband W.S. (1997–2018) ImageJ, U.S. National Institutes of Health, Bethesda, Maryland, USA. https://imagej.nih.gov/ij/.

[23] Spasova M., Mincheva R., Paneva D., Manolova N., Rashkov I. (2006). Perspectives on: Criteria for complex evaluation of the morphology and alignment of electrospun polymer nanofibers. J. Bioact. Compat. Polym..

[24] Braun E., Howard R. (1994). Adhesion of fungal spores and germlings to host plant surfaces. Protoplasma.

[25] ICDD-PDF2 # 00-021-1272. https://www.icdd.com/.

[26] Wang Y., Sun C., Zhao X., Cui B., Zeng Z., Wang A., Liu G., Cui H. (2016). The application of nano-TiO_2_ photo semiconductors in agriculture. Nanoscale Res. Lett..

[27] Ahmad N., Abdullah N., Yasin F. (2019). Antifungal activity of titanium dioxide nanoparticles against *Candida albicans*. BioRes.

[28] Ing L., Zin N., Sarwar A., Katas H. (2012). Antifungal activity of chitosan nanoparticles and correlation with their physical properties. Int. J. Biomater..

