# Supplementary material for: Electrospun Eco-Friendly Materials Based on Poly(3-hydroxybutyrate) (PHB) and TiO2 with Antifungal Activity Prospective for Esca Treatment
Source: Polymers (Basel). 2020 Jun 20;12(6):1384. doi: 10.3390/polym12061384 (PMC7361801; doi:10.3390/polym12061384)
Supplement: Supplementary file 1 [file polymers-12-01384-s001.pdf]

polymers

Supplementary material

Electrospun eco-friendly materials based on PHB and TiO_2 with antifungal activity prospective for esca treatment

Mariya Spasova[1,*], Olya Stoilova[1], Nevena Manolova[1], Iliya Rashkov[1], and Mladen Naydenov[2]

[1] Laboratory of Bioactive Polymers, Institute of Polymers, Bulgarian Academy of Sciences, Acad. G. Bonchev St, bl. 103A, BG-1113 Sofia, Bulgaria; mspasova@polymer.bas.bg; stoilova@polymer.bas.bg; manolova@polymer.bas.bg; rashkov@polymer.bas.bg

[2] Department of Microbiology, Agricultural University, BG-4000 Plovdiv, Bulgaria; mladen@au-plovdiv.bg

* Correspondence mspasova@polymer.bas.bg; Fax: +359 (0)2 8700309

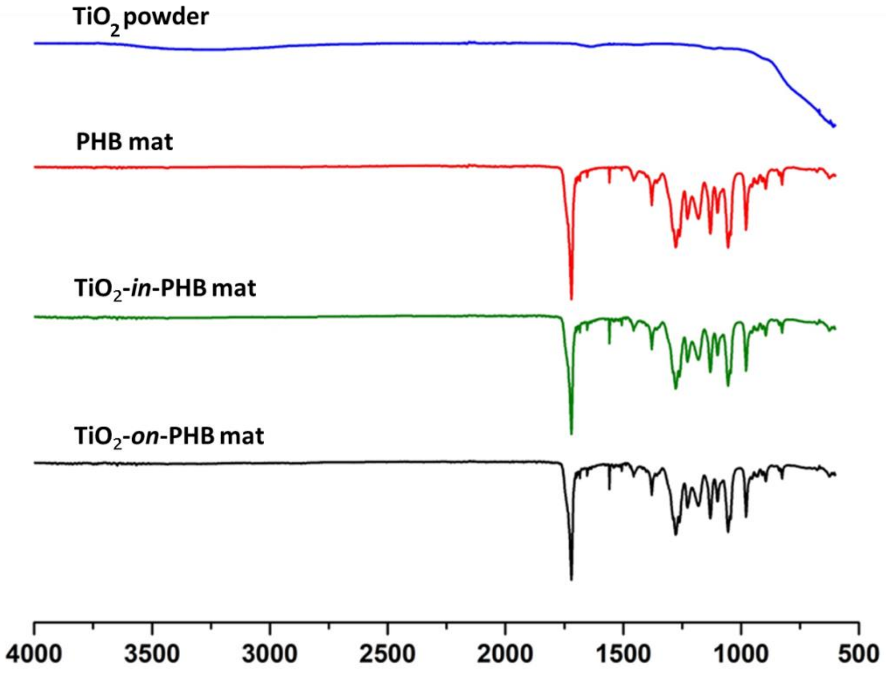

Figure S1. IR-spectra of fibrous materials and nanoTiO_2 in the range of 600–4000 cm^{-1}.
